# Using Diverse Communication Strategies to Re-Engage Relapsed Tobacco Quitline Users in Treatment, New York State, 2014

**DOI:** 10.5888/pcd12.150191

**Published:** 2015-10-22

**Authors:** Beatriz Carlini, Lyndsay Miles, Suzanne Doyle, Paula Celestino, James Koutsky

**Affiliations:** Author Affiliations: Lyndsay Miles, Suzanne Doyle, University of Washington Alcohol and Drug Abuse Institute, Seattle, Washington; Paula Celestino, James Koutsky, Roswell Park Cancer Institute, Department of Health Behavior, Buffalo, New York.

## Abstract

**Introduction:**

Most smoking cessation programs lack strategies to reach relapsed participants and encourage a new quit attempt. We used a multimodal intervention to encourage past quitline registry participants to recycle into services.

**Methods:**

We invited 3,510 past quitline participants back to quitline services, using messages consecutively delivered through Interactive Voice Response (IVR), followed by postcard and email reminders, 2 Short Messaging Services (SMS) texts, and a final cycle of IVR. The primary study outcome was recycling into a new quitline-assisted quit attempt. We used statistical analyses to assess rates and predictors of recycling (socioeconomic, health- and tobacco-related variables) with study participants and compared the study sample with registry participants not selected for the study (comparison group).

**Results:**

Quitline services were re-initiated by 12.2% of the intervention sample and 1.9% of the comparison group (z = 6.03, *P* < .001, effect size of 0.44). Most re-enrollments were done via direct IVR-transfer to the quitline. Predictors of re-enrollment were age (odds ratio [OR] = 1.45 for every 10 years of age; 95% confidence interval [CI], 1.34–1.57), number of years smoking (OR = 1.27; 95% CI, 1.18–1.36), and reporting cancer (OR = 2.32; 95% CI, 1.47–3.68) or chronic obstructive pulmonary disease (OR = 1.55; 95% CI, 1.16–2.10). Living with other smokers was correlated with a lower chance of recycling into treatment (OR = 0.72; 95% CI, 0.57–0.91).

**Conclusion:**

Recycling previous quitline participants using a proactive, IVR-based intervention is effective in reinitiating quitline-assisted quit attempts. Older, long-term smokers reporting chronic conditions are more likely than younger smokers to re-engage in quitline support when these methods are used.

## Introduction

Relapse is the most frequent outcome of people who stop smoking; typically, several quit attempts are required to achieve sustained tobacco abstinence (1). Relapsed smokers are interested in treatment but generally do not seek it proactively (2–6). Most smoking cessation programs lack strategies to encourage relapsed smokers to make another quit attempt (“recycle”). Research suggests that relapsed smokers experience decreased self-efficacy and feelings of disappointment and guilt that may hinder them from proactively seeking treatment (2). They may also have limited knowledge about treatment and health coverage for repeated quit attempts (4). Effective interventions are needed to reconnect relapsed smokers with cessation support.

Carlini and colleagues (3,4) conducted 2 randomized controlled trials that tested the feasibility and efficacy of proactive interventions inviting unsuccessful quitters to enroll in a new quitline-supported quit attempt. In the first trial, quitline staff contacted the participants by telephone. In the second trial, they used an automated telephone system (Interactive Voice Response [IVR]). In both studies, motivational and information barriers to a new quit attempt were addressed, followed by an immediate option to connect with quitline support. Recycling rates in both trials were 8 to 12 times higher among participants in the intervention group than among participants in the control group. Older smokers (aged 40 or older) were more likely to respond positively to these interventions than were younger smokers.

In this study, we tested the impact of adding Short Messaging Service (SMS) texts, email messages, and a postcard to the IVR intervention. We hypothesized that diversification of communication channels would make this intervention more effective in recycling younger smokers (aged 18–39) into a new quitline-assisted quit attempt.

## Methods

### Study design

We created a registry of 26,696 individuals who received smoking cessation support services from the New York State Smokers’ Quitline (NYSSQ [www.nysmokefree.com]) from October 1, 2012, to September 30, 2013. The registry contained data on individuals’ sociodemographic, health, and tobacco-related characteristics. Study procedures were approved by the Roswell Park Cancer Institute Institutional Review Board (IRB).

Registry´s inclusion criteria were being aged 18 or older, having received services in English, providing verbal consent to be contacted by telephone, being a cigarette smoker, not being incarcerated, and not having received quitline services for at least 5 months before the study launch.

### Study sample

We randomly selected 4,002 registry members using 3 equal-sized stratification groups according to elapsed time since initiation of previous quitline support (6–9 months, 10–13 months, and 14–17 months). Of these 4,002, we excluded 130 registry members because they re-contacted the quitline after the IRB letter was sent but before the intervention delivery started. Of the 3,872 remaining, 180 people opted out of the study, 11 had family members reporting them deceased or incarcerated, and 171 who answered the IVR call reported not smoking cigarettes in the last 30 days. The final sample comprised 3,510 participants. The participation rate was 90.6% (3,510 of 3,872). All registry members minus the original study sample (n = 4,002) served as a comparison group (n = 22,824). We sent letters to the intervention sample, describing the study and providing instructions on how to opt out (toll-free number, email, or prepaid pre-addressed letter) of the study.

In the NYSSQ standard of care, former participants may call the quitline to re-initiate support for quitting, but the quitline does not deliver interventions as part of their standard care to encourage them to do so. We delivered the intervention from April 4 to May 26, 2014, using IVR, email, SMS (ie, text messaging), and a postcard.

The intervention was delivered in 3 steps (Figure). The first step was 6 IVR attempts to reach participants (3 to each telephone number, if 2 numbers were on file) and deliver a script developed and tested in a previous study (4). After confirming that the person who answered the call was the intended recipient and was still smoking, the IVR system delivered a set of questions to identify motivational and informational barriers to recycling into a new quit attempt and provided tailored messages to specifically address these barriers. The IVR also allowed respondents to be automatically transferred to a quitline coach, record their contact information and receive a call back from the quitline, receive another call from the automated system in 2 weeks, or receive no further contact.

**Figure:**
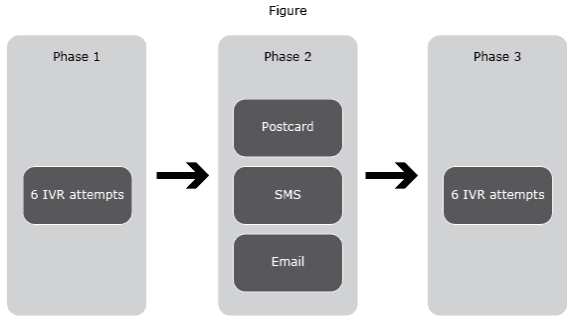
The New York State 3-phase Intervention to encourage relapsed smokers to make another quit attempt, New York State, 2014. Abbreviations; IVR, Interactive Voice Response; SMS, Short Messaging Service.

The second step targeted participants who did not answer the automated IVR calls. These participants received a postcard, an email (if they had an address on file, about 30% of the sample), and 2 SMS messages.

The email and text messages normalized relapse, reiterated that a successful quit often takes multiple quit attempts, and reminded recipients that free services were available, including nicotine replacement therapy (NRT). They could click on links in the body of the email or SMS and enroll in Web support, call the quitline, or request a call back from a quitline coach. Since the NYSSQ did not routinely document which numbers were mobile phones, SMS text messages were sent to all telephone numbers on file.

The postcard stated that the quitline was trying to reach the participant, provided the quitline number and hours, and reiterated the IVR system’s telephone number (caller identification) to encourage people who may be screening their calls. A few weeks later, a second round of 6 attempts to deliver the IVR recycling intervention was delivered to all participants who had neither been reached by IVR in the first round nor had re-contacted the quitline since study launch.

### Measures

Recycle into quitline services was obtained by consulting quitline files and is defined as a participant recontacting the quitline via telephone or internet to engage in a new quitline-supported quit attempt from April 4 to May 31, 2014 (the intervention period plus 7 days).

Sex, age, race/ethnicity, education, and chronic conditions (diabetes, asthma, cancer, depression, chronic obstructive pulmonary disease [COPD]/emphysema, and coronary artery disease [CAD]) were obtained through the registry.

Cigarettes smoked per day, time before first cigarette, years of smoking, previous quit attempts, presence of other smokers at home, and confidence and motivation to quit were obtained through the registry.

We collected 7-day abstinence and quit attempts lasting 24 hours or more at the 90-day follow-up. These secondary outcomes were assessed only among the intervention sample that recycled into quitline support. Trained personnel at Roswell Park Cancer Institute collected this information via telephone interviews. A minimum of 3 attempts were made to reach participants.

Differences between the final study sample and the comparison group of registry members were assessed with *t* tests for continuous variables (age in years and number of years smoking) and χ^2^ tests for proportions. Similar analyses were conducted to evaluate differences on these same variables between people in the study sample and in the comparison group who contacted the quitline to restart services. We used logistic regression analyses to test which of the socioeconomic- and health- and tobacco-related variables predicted recycling into a new quit attempt in the intervention sample (n = 3,510). These regression results were interpreted with odds ratios (ORs) and their corresponding 95% confidence intervals (CIs).

Initially, a multivariate regression analysis approach was attempted, using all predictor variables in the model. However, because of the high correlation between age and number of years smoking, and high correlations of these 2 variables with most of the other predictor variables, their effects were cancelled out. Therefore, we analyzed each individual predictor separately in a simple regression model to evaluate their effects. All statistical analyses were performed using SAS 9.3 software (SAS Institute, Inc).

The variable cigarettes per day was not used in the analysis because this variable is used only to determine if a smoker is eligible to receive free NRT from the NYSSQ. Only smokers who report smoking every day or almost every day were eligible to receive NRT from NYSSQ during the study period, and this information was often provided up front. Close to 100% of the registry sample reported smoking every day.

## Results

The intervention sample and comparison groups were similar in their main characteristics (Table 1). Participants were mostly in their mid-forties, were white non-Hispanic, had high school education or less, and were either publicly insured or uninsured. About a third reported at least one chronic condition.

**Table 1:** Sociodemographic and Other Characteristics of Intervention Sample and Comparison Group, New York State, 2014^a^

Variable	Study Sample (n = 3,510)	Comparison Group (n = 22,824)	*P* Value
**Mean age, y (SD) (effect size = 0.14)**	45.9 (14.1)	47.8 (13.9)	<.001
**No. years smoking (SD) (effect size = 0.09)**	22.8 (14.3)	24.1 (14.9)	<.001
**Sex**			
Female	53.8	54.0	.79
**Age, y, %**			
<40	34.8	28.9	<.001
≥40	65.2	71.1	
**Insurance**			
Uninsured	26.8	29.0	.16
Medicaid	38.1	35.2	.05
Medicare	11.4	12.5	.52
Private	23.7	23.2	.85
**Race/ethnicity**			
White, non-Hispanic	65.6	65.3	.61
**Education**			
High school or less	61.7	61.2	.61
**Selected smoking variables**			
1st cigarette within 5 min	54.3	54.1	.85
Tried to quit before	78.4	79.4	.21
Live with other smokers	31.3	30.8	.60
**Chronic conditions**			
Depression	16.0	15.7	.20
Asthma	12.7	11.9	.12
Emphysema/COPD	10.9	10.6	.60
Diabetes^b^	7.4	8.5	.03
Heart disease	3.5	3.6	.98
Cancer	3.0	3.3	.33
**Importance of quitting**			
Not at all	0.2	0.2	.62
Somewhat	7.5	7.5	
Extremely	92.3	92.4	
**Confidence in ability to quit**			
Not at all	1.3	1.3	.91
Somewhat	19.8	19.5	
Extremely	78.9	79.3	

Abbreviations: COPD, chronic obstructive pulmonary disease; SD, standard deviation.

a Values are expressed as percentages unless otherwise indicated.

b The effect size for diabetes is 0.04.

The final study sample (n = 3,510) and the comparison group (n = 22,824) differed significantly only in terms of mean age, number of years smoking, and proportions with diabetes (Table 1). The effect sizes of these differences were relatively small (0.14, 0.09, and 0.04, respectively).

A total of 859 people contacted the quitline to recycle into services during the study period. The intervention sample had a higher proportion of new contacts (12.2%, n = 429) than the comparison group (1.9%, n = 430). This difference in proportions was significant (z = 6.03, *P* < .001), with an effect size of 0.44. There were no significant differences between the 2 groups in terms of demographics, health, tobacco-related characteristics, and reported importance of and confidence in being able to quit (Table 2).

**Table 2:** Sociodemographic, Health, and Tobacco Use Characteristics of People Who Re-Initiate a Cycle of Quitline-Supported Quit Attempt, by Study Sample and Comparison Group, New York State, 2014

Characteristic	Study Sample Registry (N = 429)	Comparison Group (n = 430)	*P* Value
**Age, mean (SD), y**	52.2 (12.1)	22.8 (14.3)	.27
**No. of years smoking**	51.2 (13.2)	24.1 (14.9)	.29
**Sex**			
Female	50.6	56.0	.11
**Race/ethnicity**			
White, non-Hispanic	66.8	65.7	.72
**Education**			
High school or less	55.9	58.7	.46
**Selected smoking variables**			
1st cigarette within 5 min	52.8	56.6	.28
Tried to quit before	82.8	79.3	.19
Live with other smokers	25.5	26.8	.65
**Chronic conditions**			
Depression	16.8	17.7	.72
Asthma	12.2	12.8	.82
Emphysema/COPD	15.1	13.0	.38
Diabetes	7.8	8.1	.85
Heart disease	3.5	2.8	.53
Cancer	5.9	4.4	.33
**Importance of quitting**			
Not at all	0	0	.38
Somewhat	5.8	4.4	
Extremely	94.2	95.6	
**Confidence in ability to quit**			
Not at all	1.8	0.8	.41
Somewhat	17.8	17.0	
Extremely	80.5	82.3	

Abbreviations: COPD, chronic obstructive pulmonary disease; SD, standard deviation.

Certain variables significantly predicted recycling into a new quitline-supported quit attempt in the intervention sample (Table 3). In the bivariate analysis, age was the most significant predictor; every 10 years’ increase in age resulted in a 45% increase in the odds of recycling into quitline support. Furthermore, analyses could not support the hypothesis that diversification of communication channels would make this intervention more effective in recycling younger smokers into a new quit attempt. In contrast, a logistic regression model for predicting recycling indicated that the odds of those aged 40 years or older to recycle is at least 3 times that of those younger than 40. Similarly, every 10 years’ increase in the number of years smoking resulted in a 27% increase in the odds of recycling.

**Table 3:** Significant Predictors of Recycling Into a New Quitline-Supported Quit Attempt Among 3,510 Former Users of Quitline Support^a^, New York State, 2014

Variables	OR (95% CI)	*P* Value
**Bivariate analyses**		
Age (per every 10 years)	1.45 (1.34–1.57)	<.001
No. of years smoking (per 10 years)	1.27 (1.18–1.36)	<.001
Aged 40 or older	3.08 (2.35–4.02)	<.001
Cancer diagnosis	2.32 (1.47–3.68)	.003
Emphysema/COPD diagnosis	1.55 (1.16–2.10)	.003
Tried to quit before^b^	1.37 (1.05–1.79)	.02
Greater than high school education	1.28 (1.03–1.58)	.03
Living with other smokers	0.72 (0.57–0.91)	.01
**Multivariate analysis^c^**		
Cancer	1.93 (1.16–3.23)	.02
Emphysema/COPD	1.45 (1.06–1.98)	.02
Tried to quit before^b^	1.39 (1.03–1.86)	.03
Greater than high school	1.21 (0.97–1.50)	.10
Live with other smokers	0.71 (0.56–0.91)	.01

Abbreviations: CI, confidence interval; COPD, chronic obstructive pulmonary disease; OR, odds ratio.

a Predictors that were not significant were sex, race/ethnicity, time before first cigarette, number of cigarettes per day, diabetes, heart disease, depression, asthma, confidence, motivation, and length of previous quitline service.

b This information was collected when study participants initiated the first contact with the quitline.

c Age and years smoking were eliminated from consideration in the multivariate logistic analysis, which was conducted with the remaining predictors (see Results).

People who reported having cancer (OR = 2.32) or COPD/emphysema (OR = 1.55) were also more likely to recycle. Additionally, people with more education had higher odds of recycling (OR = 1.28) than people with high school education or less. Participants who lived with other smokers had a lower chance of recycling into treatment than those who did not live with other smokers.

We also conducted a multivariate logistic analysis eliminating the variables “age” and “years smoking.” The multivariate logistic model (Table 3) indicated similar results to the bivariate analyses on the remaining 5 in terms of significance and ORs. However, in the presence of other predictors, the education level variable was no longer significant (*P* = .10) in this model.

More than half of quitline re-enrollment occurrences (58.3% or 250 of 429) were the result of a direct transfer from the IVR system to the quitline services. Most of those (69.8%) recycled in services during the first round of call attempts (Figure). Only 35 people (8%) reinitiated quitline support during the email and SMS interventions. It was not possible to establish a direct connection between recycling occurrences and the email and SMS interventions, because there was no mechanism to identify which quitline re-enrollments were due to a participant clicking a link on the messages provided.

The follow up rate was 53.0% (229 respondents of 429); of those, 79.9% (n = 183) reported making a quit attempt lasting 24 hours or more in the last 90 days, while 24.5% (n = 56) reported abstaining from tobacco in the last 7 days. When all nonrespondents were considered to be smokers (intent to treat), quit attempt and quit rates were 42.6% and 13.1%, respectively.

## Discussion

This study documents that quitline registries can be used to continuously support smokers in a new quit attempt. It also strengthens previous findings that proactive interventions to recycle smokers into a new quit attempt are effective (3,4). Our results indicate that older, long-term smokers are the most interested in recycling (4) and that automated telephone messages (ie, IVR) are effective when compared with standard of care (no action to proactively reach former quitline participants).

Our study found that SMS text messaging, postcards, and email communication generated few re-enrollments and did not change the demographics of smokers who recycled into quitline services. This finding suggests that low re-enrollment of younger smokers (aged 18–39) may not be related to the communication channels used — they may be less interested in using the quitline repeatedly. It is also possible that IVR, SMS, and emails are not optimal ways to reach them. TIPS, a national media campaign using television and radio ads (9) occurred right before our study and generated a 132% increase in quitline calls nationwide. Preliminary analysis suggests augmented effects among young smokers (9). Young smokers who had already used quitline services may have responded to the TIPS campaign and not to our recycling messages. Future research on the TIPS campaign effects on previous quitline users might shed some light on this possibility.

Our study also found that time elapsed since last quitline-assisted quit attempt was not a predictor of recycling into quitline services. Smokers who had tried to quit tobacco as recently as 6 months before our intervention were open to a new quitline-assisted quit attempt and did not differ from those who had last talked to the quitline more than 1 year prior. This finding has practical implications, because some quitlines require that smokers who do not quit or relapse after receiving services must wait for 12 months to be eligible for quitline services again. This embargo period for re-enrollment in quitline services should be discouraged because it prevents motivated smokers from using a quitline’s evidence-based support to make a quit attempt.

In this study, the IVR calls displayed a telephone number but not a name in the caller ID, because IVR companies who provide services to third parties are not able to customize them. About 30% of smokers who recycled into quitline support through IVR calls did so only after we mailed them postcards reiterating the IVR number, although we attempted to reach them 6 times before. Because many people screen calls, the postcard was probably associated with more calls being answered. Another possibility is that sending postcards, SMS, and email messages concomitantly (Figure) worked synergistically, generating willingness to answer the IVR call and re-initiate quitline support after its delivery.

Smokers reporting cancer or COPD/emphysema were more likely to recycle into quitline support than other smokers. This finding is consistent with those of previous studies (4,10,11). A recent secondary analysis using interview waves of older adults also found that the odds of smoking cessation increased with a new diagnosis of chronic illness (12). Clinicians should be made aware that patients with smoking-related illnesses may be interested in receiving support to quit, even if they continue to smoke after being advised to quit.

The main strengths of this study — using a registry of past quitline users and conducting a study in the real world of a state quitline — are also the source of some limitations. First, the study was conducted in a registry of quitline past users, or in other words, among people who reported being smokers 6 to 17 months before the study launched. We could not ascertain the current smoking status of all participants in the study. Although this limitation prevented us from knowing the true denominator of our intervention, it also likely mimics how quitline services would use their limited resources if they were to reach their former participants for recycling interventions.

A second limitation was that the SMS intervention was sent to telephone numbers that may not have been associated with mobile devices capable of receiving text messages. Third, only about one-third of participants had email addresses on file, limiting the reach of the intervention. However, because only 8% of those who recycled into quitline support called during the 2 weeks of the SMS and email interventions, it seems unlikely that we would obtain significantly different results by restricting our study eligibility criteria to participants with email addresses and type of telephone documented.

A fourth limitation is related to not conducting a randomized clinical trial. Using a comparison group much larger than the intervention arm was not ideal; therefore, we decided to report on effect size of the significant differences detected.

A fifth limitation was that we could not analyze past insurance status accurately, because NYSSQ’s recording system updates insurance status by overwriting the status every time a participant contacts the quitline. This practice, coupled with the unusually high level of health insurance changes during fiscal year 2013–14 due to the Affordable Care Act (7), compromised the validity of this variable for this study. 

Quitline registries can be used to encourage smokers to engage in a new quit attempt. Telephone-based proactive contact with previous users of quitline services is an effective way to re-initiate a new quitline-assisted quit attempt. Older, long-term smokers who report chronic conditions are more likely than younger smokers to engage in a new cycle of quitline support. More research is needed to identify barriers and test interventions that motivate younger smokers to recycle in cessation support.
